# Surgical ductal stent implantation in total anomalous pulmonary venous connection to vena porta with right pulmonary sequestration in a mature newborn

**DOI:** 10.1016/j.amsu.2019.07.005

**Published:** 2019-07-11

**Authors:** Huseyin Avni Solgun, Tugcin Bora Polat

**Affiliations:** a Department of Pediatrics, Altınbas University Medical Park, Bahcelievler Hospital, Istanbul, Turkey; b Department of Pediatric Cardiology, Altınbas University Medical Park, Bahcelievler Hospital, Istanbul, Turkey

**Keywords:** Total anomalous pulmonary venous connection, Pulmonary sequestration, Ductal stent implantation

## Abstract

In many cases of total anomalous pulmonary venous connection (TAPVC), the four pulmonary veins (PV) join together behind the left atrium, where they form a collector. This collector can drain into the right atrium directly through the innominate vein into the superior vena cava (SVC), into the coronary sinus (CS), or through the diaphragm to the venous structures of the abdomen. In our case, a mature newborn had TAPVC draining into the vena porta along with severe pulmonary hypertension. Additionally, there were right pulmonary sequestration, dextrocardia, transposition of the great arteries, severe pulmonary stenosis, and single ventricular pathology in echocardiographic examination. Clinical signs manifested in the first 7 days of life. Diagnostic tools used were echocardiography and angiography. A ductal stent was surgically implanted into the ductus arteriosus by angiography. TAPVC was found to be nonobstructive. Therefore, we would like to emphasize the rareness and hardness to perform the surgical ductal implant technique in our particular case of TAPVC with pulmonary sequestration draining into the vena porta. The prognosis in TAPVC is poor and related mainly to the existence of pulmonary venous obstruction.

## Highlights

• In many cases of total anomalous pulmonary venous connection (TAPVC), the four pulmonary veins (PV) join together behind the left atrium, where they form a collector.
• In our case, a newborn was diagnosed with total anomalous pulmonary venous connection (TAPVC) draining into the vena porta along with severe pulmonary hypertension. Additionally, there was right pulmonary sequestration, dextrocardia, transposition of the great arteries, severe pulmonary stenosis, and single ventricular pathology in echocardiographic examination.
• We would like to emphasize the rareness of the anatomical structure and hardness of the surgical technique in our particular case of TAPVC with additional pulmonary sequestration draining into the vena porta.

## 1 Case

**Fig. 1:**
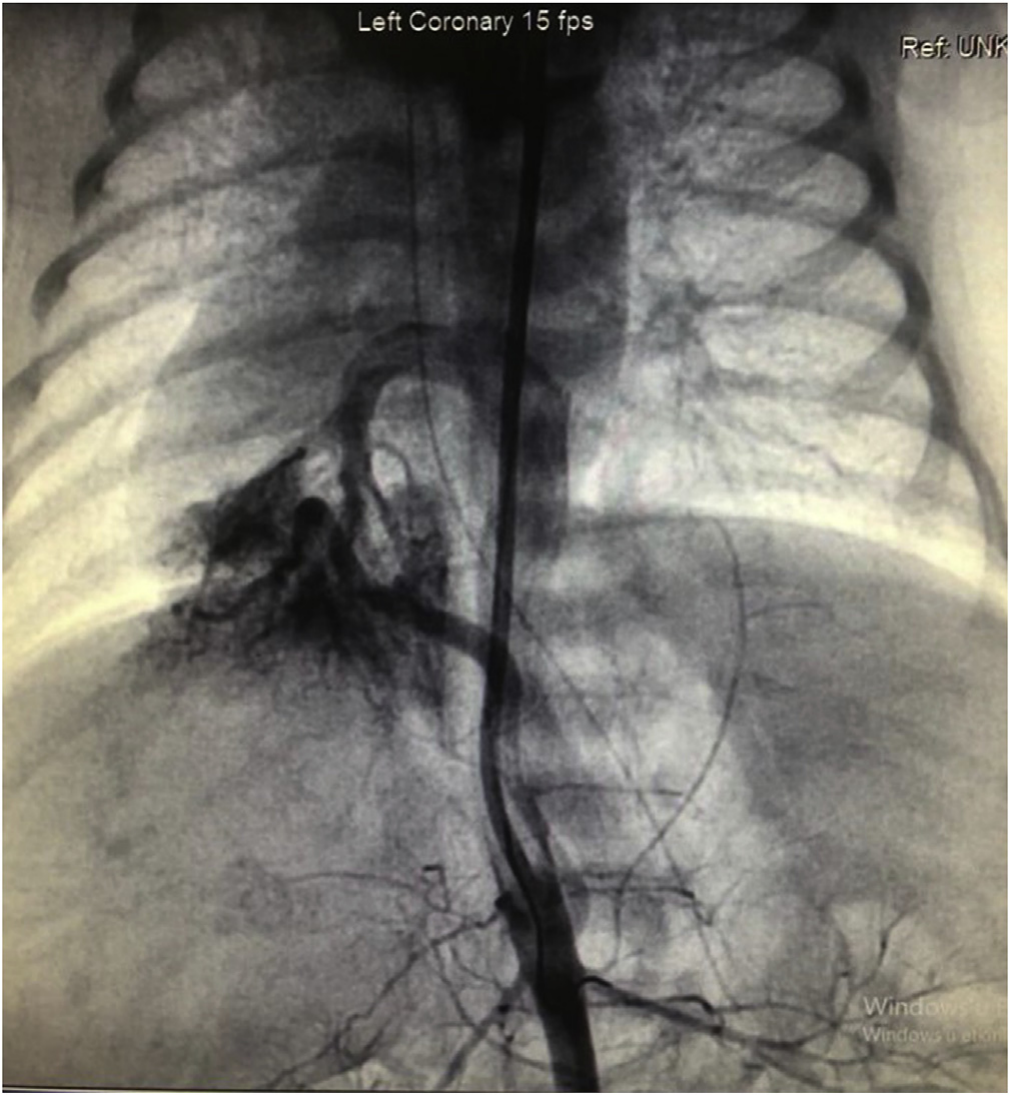
Major aortopulmonary collateral arteries (MAPCAs) supply from the abdominal aorta draining into the common long chamber of the pulmonary veins due to sequestration of pulmonary arteries.

**Fig. 2:**
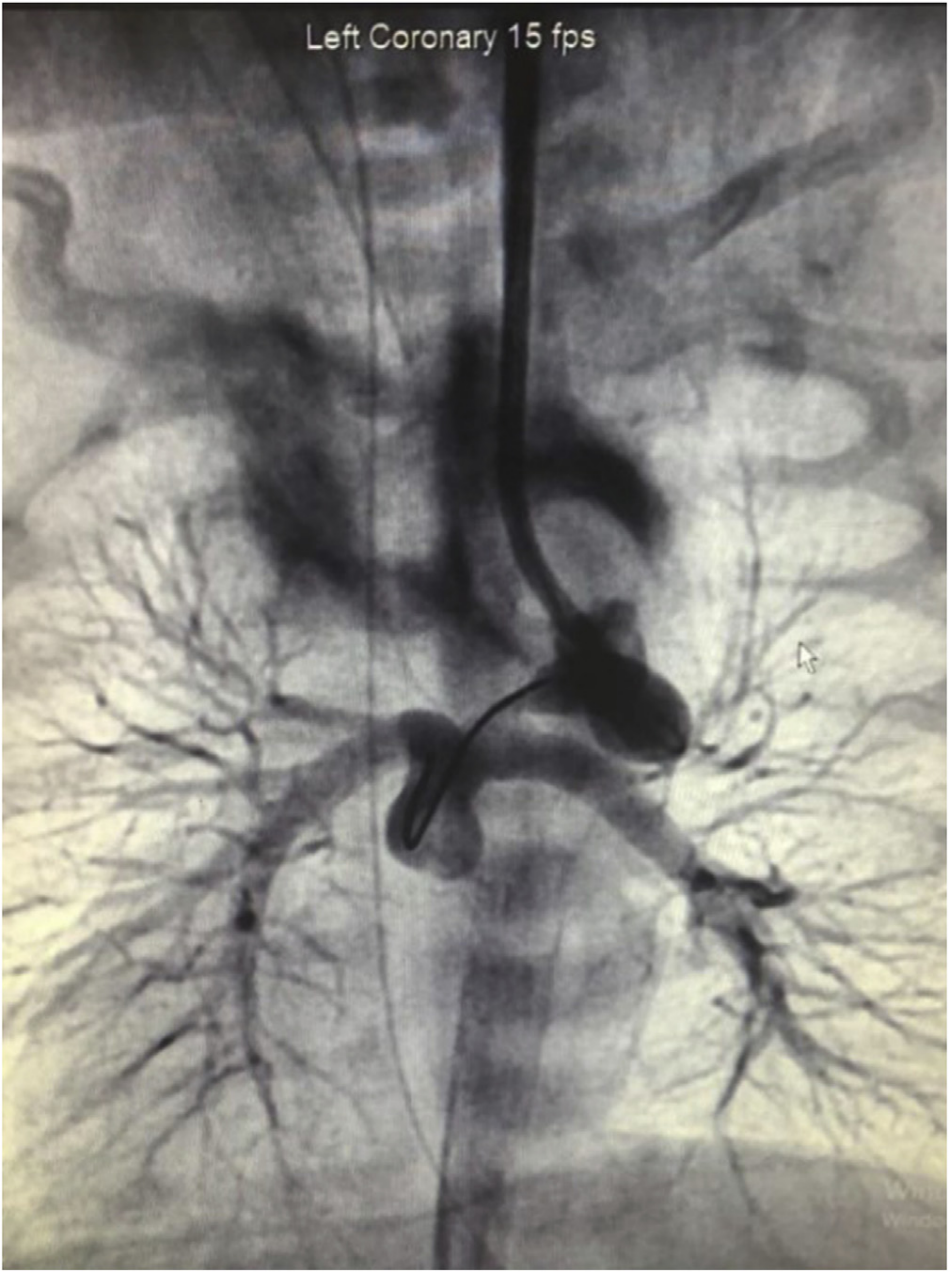
View of the pulmonary veins draining into the portal system through a common long chamber.

**Fig. 3:**
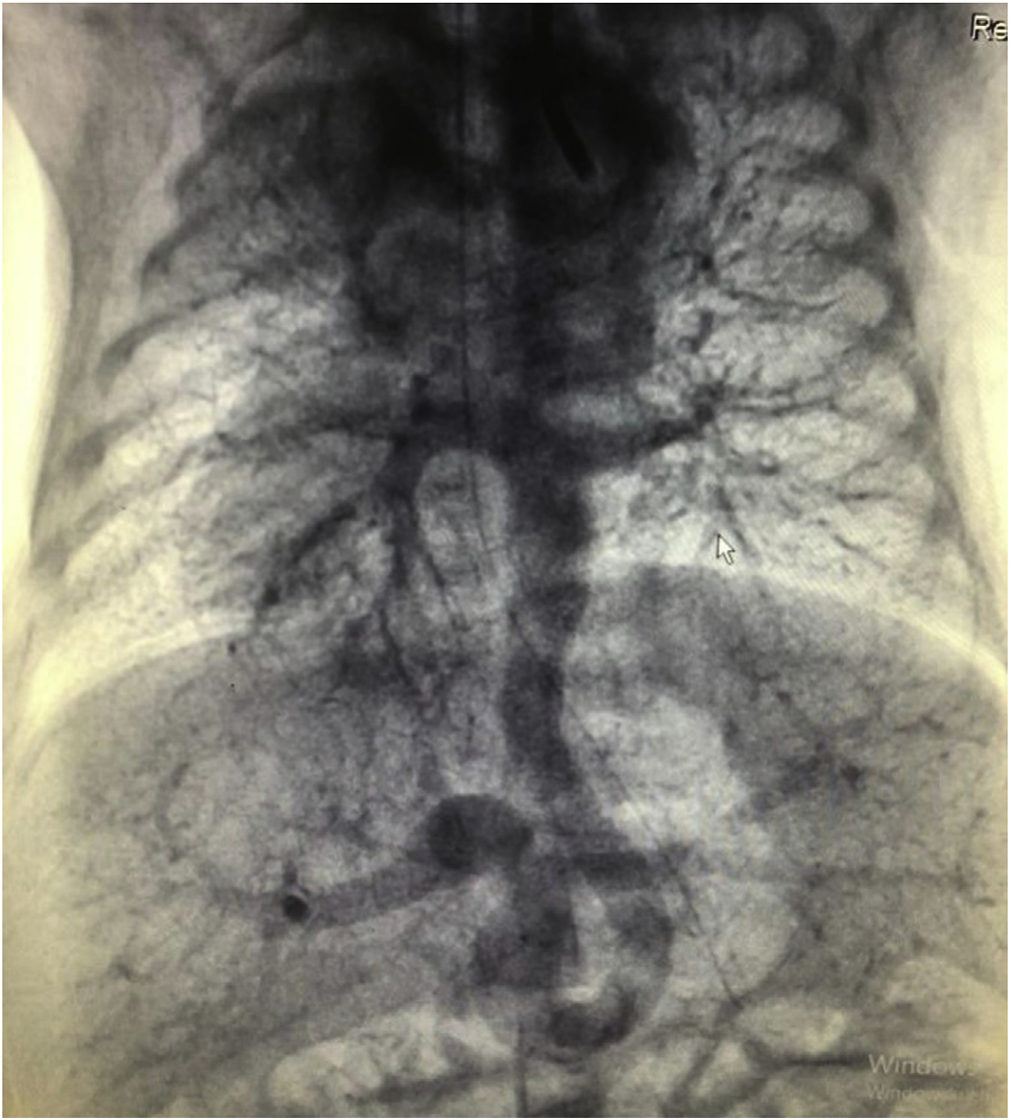
Ductal stent of ductus arteriosus through the carotid artery due to severe pulmonary stenosis and cyanosis.

A mature newborn patient at gestational age of 39 weeks born by cesarean section delivery in another hospital was referred to our hospital because of the intrauterine diagnosis of TAPVC by fetal echocardiography. The following preoperative data were recorded: body weight was 2820 g, and height was 50 cm. On physical examination, the general condition was severe, moro reflex was hypoactive, respiration rate was 55 breaths per minute, heart rate was 128 beats per minute; during auscultation of the heart, there were 2–3/6 systolic murmurs; blood pressure was 78/37 mmHg, oxygen saturation <65%, and capillary refill was 3 seconds. During patient follow-up, the electrocardiogram (ECG) showed ST-segment elevation of 2 mm in the VII and aVF leads. An echocardiogram was recorded, which showed TAPVC, right pulmonary sequestration, dextrocardia, transposition of the great arteries, pulmonary stenosis, and single ventricular pathology (Fig. 1)***.*** The staff of the previous hospital had started prostacyclin infusion to continue ductal flow due to cyanotic congenital heart diseases. On the fourth day of life, coronary angiography was performed, and a ductal stent was implanted into the ductus arteriosus through surgery (Fig. 2)***.*** The ductal stent of ductus arteriosus was implanted through the carotid artery to treat severe pulmonary stenosis and cyanosis (Fig. 3)***.*** Postoperative findings showed a respiration rate of 42 breaths per minute, heart rate of 110 beats per minute, blood pressure of 75/35 mmHg, oxygen saturation of >90%. Assessment of the clinical status showed to be more stable than before.

This study have been issued due to SCARE 2018 Statement; Updated Consensus Surgical Case Report (SCARE) Guidelines creterias [1]

## 2 Discussion

From the embryologic standpoint, the pulmonary venous plexus engages the splanchnic plexus and communicates with cardinal veins and the umbilicovitelline system. The CPV grows from the left atrium and connects with the pulmonary venous plexus. This direct connection allows atrophy of primitive complex systemic venous channels and provides normal pulmonary venous drainage into the left atrium through four central parts of the pulmonary vein (right upper, right lower, left upper, and left lower CPPV) [[2], [3], [4]]. It is believed that total anomalous pulmonary venous return results from abnormal development of the CPV and persistence of the embryologic pulmonary–systemic venous anastomosis [2]. TAPVC is explained by the atresia of the common pulmonary vein, while a communication between the venous splanchnic and pulmonary plexus still exists. Collateral arteries persist in the primitive form, and their presence can generate TAPVC through abnormal myocardialization and smooth muscle cell formation [5].

In some cases, autosomal dominant inheritance has been suspected, but the number of families in whom this has been suspected is very few – 11 families [6]. Exposure to painting or paint stripper, lead, and pesticides during the first three months of pregnancy has been associated with TAPVC in the Baltimore–Washington Infant Study [7]. Syndromes such as Holt-Oram, Klippel-Feil, and phocomelia may be associated with TAPVC.

Scimitar syndrome (SS) is a syndrome characterized by specific features: partial or total right anomalous pulmonary venous return to the inferior vena cava (IVC), right pulmonary artery hypoplasia, atrial septal defect (ASD), right lung hypoplasia, aortopulmonary collateral vessels, diaphragmatic hernia, and horseshoe lung [8]. The SS sign is seen in partial anomalous pulmonary venous drainage of the right lower lobe. The anomalous vein is curvilinear and parallels the right heart border to drain into the intra-thoracic inferior vena cava. Its appearance on the chest radiograph is likened to the scimitar, a type of sword characterized by its curved shape. Common associations include congenital heart disease and pulmonary hypoplasia.

Pulmonary sequestration is a congenital anomaly of the primitive foregut. The most plausible theory is the formation of an accessory supernumerary lung bud below the normal lung bud [9]. It continues to migrate caudally with the esophagus in extralobar sequestration and then derives its blood supply from the primitive splanchnic vessels surrounding the foregut [10]. Associated congenital anomalies occur more frequently with extralobar sequestration than with intralobar sequestration. Approximately 50%–60% of patients have another congenital anomaly. It may include congenital diaphragmatic hernia, congenital cystic adenomatoid malformation, vertebral defect, congenital heart disease, tracheoesophageal fistula, pulmonary hypoplasia, bronchogenic cyst, and congenital megacolon. A congenital diaphragmatic hernia is the commonest [11]. In literature, co-existence of TAPVC and pulmonary sequestration is rare and nearly is not shown.

In our case, we presented a very rare co-existence of TAPVC draining into the vena porta along with right pulmonary sequestration, dextrocardia, transposition of the great arteries, pulmonary stenosis, and single ventricular pathology, all of which are not usual and common in SS. In literature, this co-existence is shown only in SS with partial anomalous pulmonary venous drainage of the right lower lobe but not in other complex cardiac pathologies like in our case. Additionally, it is considerable to point the hardness of ductal stent implantation in such a case. For this point of view, our case is important to emphasize this issue.

## 3 Conclusion

TAPVC with pulmonary sequestration is a rare entity. In complex cardiac pathologies of congenital heart diseases, surgical treatment options have to be evaluated carefully for each case to obtain better outcomes.

## Ethical approval

Altınbas unıversity ethical committie have given approvel for the study.

Medicine faculty dean Prof. MD Tunc Fısgın is the resposable of the approvel.

There is no Judgement reference number, sign manually by commitie members.

## Sources of funding

None.

## Author contribution

Writer Huseyin Avni Solgun have made study design, writing and data analysis.

Writer Tugcin Bora Polat have made data collection and data analysis.

## Conflicts of interest

None declared.

The patients parents (Both dad and mom) has given consent for possible publication of this case report.

## Trial registry number – ISRCTN

None.

## Guarantor

Asistant Proffessor MD Huseyin Avni Solgun.

## Research registration Unique Identifying Number (UIN)

None.

## Provenance and peer review

Not commissioned, externally peer reviewed.

## Consent from patients

The patients’ parents have given consent for possible publication of this case report.
